# A randomized controlled comparison of sublay and onlay mesh techniques in ventral abdominal wall hernia repair

**DOI:** 10.12669/pjms.42.(ICON26).15688

**Published:** 2026-04

**Authors:** Sana Ehsan Ullah, Muhammad Irshad Hussain, Irfan Javed

**Affiliations:** 1 Sana Ehsan Ullah, FCPS General Surgery. Senior Registrar, Department of General Surgery, Recep Tayyip Erdogan Hospital, Muzaffargarh, Pakistan; 2 Muhammad Irshad Hussain, FCPS, FRCS, FEBS. Consultant General Surgeon and Head Department of General Surgery, Recep Tayyip Erdogan Hospital, Muzaffargarh, Pakistan; 3 Irfan Javed, FCPS General Surgery. Consultant General Surgeon, Department of General Surgery, Recep Tayyip Erdogan Hospital, Muzaffargarh, Pakistan

**Keywords:** Mesh repair, Onlay, Recurrence, Seroma, Sublay, Ventral hernia, Wound infection

## Abstract

**Objective:**

To compare wound infection, seroma formation, and hernia recurrence rates between onlay and sublay mesh repair for ventral abdominal wall hernias.

**Methodology:**

This prospective, randomized controlled trial was conducted at Recep Tayyip Erdogan Hospital, Muzaffargarh, Pakistan, from February 2022 to August 2023. Ninety-six patients aged 18–60 years with ventral hernias (defect size 40–100 mm) were randomly assigned to onlay or sublay mesh repair (48 per group). Outcomes—wound infection, seroma, and recurrence—were evaluated during hospitalization and at two week and six month follow-ups. Data were analyzed using SPSS v26.0; *p* ≤ 0.05 was considered significant.

**Results:**

The mean age was 43.4 ± 8.8 years in the onlay group and 44.5 ± 11.2 years in the sublay group (*p* = 0.592). Wound infections occurred in 22.9% of onlay versus 8.3% of sublay patients (*p* = 0.049). Seroma formation occurred in 8.3% versus 4.2% (*p* = 0.678), and recurrence in 0% versus 2.1% (*p* = 0.495), respectively. In defects 5.1–10 cm, infection was significantly higher in onlay repairs (37.5% vs 12%; *p* = 0.03).

**Conclusion:**

Sublay mesh repair significantly reduced wound infections compared with onlay repair, with comparable seroma and recurrence rates, supporting its preference in resource-limited settings.

Registration No.ClinicalTrials.gov (NCT07140471).

## INTRODUCTION

Ventral abdominal wall hernias, including primary (umbilical, epigastric, paraumbilical) and incisional types, represent a major surgical burden worldwide, with an estimated two to three million repairs performed annually.^1^ In the United States, around 350,000 repairs are performed each year, adding more than $3.2 billion to healthcare costs.^2^ In Pakistan, the prevalence of ventral hernias is rising due to increasing obesity, frequent abdominal surgeries such as cesarean sections, and limited access to timely surgical care—trends consistent with global observations.^3^,^4^ Risk factors including obesity (BMI >30 kg/m²), diabetes, smoking, and postoperative wound infection contribute to hernia formation and recurrence.^5^

In resource-limited regions, open mesh repair remains the mainstay of treatment because access to advanced imaging and laparoscopic facilities is often restricted.^6^ The introduction of prosthetic mesh has markedly improved outcomes; however, the optimal placement plane—onlay (over the anterior rectus sheath) or sublay (retromuscular) remains debated.^7^ The onlay technique is technically simpler but requires wider subcutaneous dissection, predisposing to wound complications, whereas sublay repair offers better mesh integration and lower infection risk but demands greater surgical expertise.

Although mesh hernia repair is generally classified as a clean procedure, infection rates are typically below five percent in optimal settings but may be higher in developing countries.^8^ Seroma formation, reported in five (25%) of cases, is influenced by mesh size, defect size, and surgical technique.^9^ Recurrence rates range between one–10%, depending on patient factors and follow-up duration.^10^ Laparoscopic repair offers smaller incisions, lower wound complications, and faster recovery but remains limited in low- and middle-income countries due to cost and infrastructure constraints.^11^ Consequently, open mesh repair continues to be the standard approach in such settings.

Despite several international studies, prospective local evidence comparing onlay and sublay mesh repair in Pakistan is scarce. Most available data are retrospective or small-scale. This randomized controlled trial (RCT) was conducted to compare wound infection, seroma formation, and recurrence between onlay and sublay mesh repair, providing evidence applicable to resource-limited environments.

## METHODOLOGY

This prospective, open-label randomized controlled trial was conducted in the Department of General Surgery, Recep Tayyip Erdoğan Hospital, Muzaffargarh, Pakistan, from February 2022 to August 2023. A total of 96 adult patients with ventral abdominal wall hernias were enrolled. The sample size (n = 96; 48 per group) was calculated using OpenEpi version 3.0, assuming a 20% difference in wound infection rates between groups, 80% power, a two-sided α of 0.05, and a 10% attrition rate. Participants were randomized in a 1:1 ratio using a computer-generated random number sequence into:

- **Group-A:** Onlay mesh repair (n = 48)
- **Group-B:** Sublay mesh repair (n = 48)

Group-A comprised 24 males and 24 females with a mean age of 43.41 ± 8.83 years, while Group-B included 24 males and 24 females with a mean age of 44.52 ± 11.17 years. Baseline demographic and clinical characteristics were comparable between the two groups (Table-I). Allocation concealment was ensured using sequentially numbered, sealed opaque envelopes. Due to the nature of the intervention, blinding of surgeons and participants was not feasible; however, outcome assessors and data analysts were blinded to group allocation.

**Table-I:** Patient Characteristics.

Variable	Total (N=96)	Onlay (N=48)	Sublay (N=48)	p-value
Age (years)	43.96 ± 10.03	43.41 ± 8.83	44.52 ± 11.17	0.592
BMI (kg/m²)	27.08 ± 3.42	27.44 ± 3.47	26.72 ± 3.37	0.300
Defect Size (cm)	5.70 ± 1.66	5.61 ± 1.62	5.79 ± 1.70	0.565
Gender (M/F)	48/48	24/24	24/24	1.000
Smokers	16 (16.7%)	9 (18.8%)	7 (14.6%)	0.589

### Ethical statement:

Ethical approval was obtained from the Institutional Review Board (IHHN_IRB_2023_05_026, May, 2023). The trial was registered at ClinicalTrials.gov (NCT07140471). Written informed consent was obtained from all participants prior to enrollment.

### Inclusion criteria:

- Adults aged 18–60 years
- Primary or incisional ventral abdominal wall hernia
- Hernia defect size between 40 and 100 mm
- Hernia duration of at least six months
- Diagnosis confirmed by clinical examination and ultrasonography

### Exclusion criteria:

- Diabetes mellitus of more than five years’ duration
- Chronic liver disease
- Obstructed or strangulated hernia
- Immunosuppression
- Recurrent anterior abdominal wall hernia (previous repair at the same site)
- Any contraindication to surgery

All procedures were performed by consultant general surgeons with more than five years of operative experience. Hernia defect size was categorized as ≤5 cm (n = 52) and >5 cm (n = 44). Mesh size was selected to provide at least a four–five cm overlap beyond the defect margins in all directions: 10 × 10 cm mesh for defects ≤5 cm and 15 × 15 cm mesh for defects >5 cm.

In the onlay group, the hernia sac was reduced and a sterile macroporous polypropylene mesh was placed over the anterior rectus sheath and secured with non-absorbable polypropylene sutures (2-0). Subcutaneous tissue and skin were closed with absorbable sutures.

In the sublay group, the retrorectus space was developed and an identical polypropylene mesh was placed beneath the rectus abdominis muscle and fixed using polypropylene sutures. A closed-suction drain was placed in patients with defects >5 cm in both groups and removed once output was <30 mL in 24 hours.

Postoperative management included sterile wound dressings and standard analgesia (intravenous diclofenac sodium 75 mg in 100 mL normal saline followed by oral paracetamol 1g every eight hours as required).

Primary outcomes were wound infection, seroma formation, and hernia recurrence. Wound infection was diagnosed according to Centers for Disease Control and Prevention (CDC) criteria, defined as fever >38°C, erythema, swelling, or purulent discharge occurring within 30 days of surgery. Seroma was defined as subcutaneous fluid collection confirmed by ultrasonography, while recurrence was defined as reappearance of hernia within six months confirmed clinically and by ultrasonography.

Secondary outcomes included operative time, length of hospital stay, and postoperative pain assessed using the visual analog scale (0–10). Follow-up was conducted daily during hospital stay and at two weeks and in nine months postoperatively. Ultrasonography was performed when seroma or recurrence was suspected.

Data were analyzed using SPSS version 26.0 (IBM Corp., Armonk, NY, USA). Continuous variables were expressed as mean ± SD and compared using independent-sample t-tests. Categorical variables were expressed as frequencies and percentages and analyzed using chi-square or Fisher’s exact test as appropriate. Stratification was performed by age, gender, BMI, smoking status, and defect size. A p-value ≤0.05 was considered statistically significant.

## RESULTS

A total of 96 patients were included in the study and randomized equally into the onlay group (n = 48) and the sublay group (n = 48). Baseline demographic and clinical characteristics, including age, gender, BMI, and hernia defect size, were comparable between the groups (Table-I).

Of the total participants, 52 patients (54.2%) had defect sizes ≤5 cm, while 44 patients (45.8%) had defects >5 cm. Mesh dimensions were selected to provide at least a 4–5 cm overlap beyond the defect margins: making it at least 10 × 10 cm mesh for defects ≤5 cm and 15 × 15 cm mesh for defects >5 cm. Closed-suction drains were placed in 34 patients (35.4%), 18 in the onlay group and 16 in the sublay group—primarily for larger defects (>5 cm).

### Primary outcomes:

Among the primary outcomes, summarized in Table-II, a statistically significant difference was observed only in wound infection rates between the two groups. Wound infection occurred in 11 patients (22.9%) in the onlay group and four patients (8.3%) in the sublay group (*p* = 0.049). In contrast, no significant differences were found in seroma formation, observed in four patients (8.3%) in the onlay group and two patients (4.2%) in the sublay group (*p* = 0.678)—or in recurrence, noted in one patient (2.1%) in the sublay group and none in the onlay group (*p* = 0.495).

**Table-II:** Primary Outcomes.

Outcome	Onlay (N=48)	Sublay (N=48)	p-value
Wound Infection	11 (22.9%)	4 (8.3%)	0.049
Seroma Formation	4 (8.3%)	2 (4.2%)	0.678
Recurrence	0 (0%)	1 (2.1%)	0.495

Stratified analyses for age, gender, BMI, and hernia defect size were done. No significant associations were found between the surgical technique and outcomes when stratified by age or BMI (all p > 0.05). Gender stratification showed a significant difference in wound infection rates among female patients (25% in the onlay group vs 7.9% in the sublay group; p = 0.046), whereas no difference was observed among male patients. Stratification by hernia defect size demonstrated that patients with defects >5 cm had significantly higher wound infection rates in the onlay group (37.5%) compared with the sublay group (12%) (p = 0.03). No significant differences were found in patients with smaller defects (≤5 cm). (*p* = 0.42). The presence or absence of a drain did not significantly affect wound infection or seroma rates in either group (*p* > 0.05).

### Secondary outcomes:

Among the secondary outcomes, presented in Table-III. a statistically significant difference was observed only in the mean operative time, which was shorter in the onlay group (72.3 ± 12.1 minutes) compared with the sublay group (78.6 ± 14.3 minutes) (*p* = 0.032). In contrast, no significant differences were found in the mean duration of hospital stay (4.2 ± 1.1 days vs 4.0 ± 0.9 days, *p* = 0.318) or in postoperative pain at two weeks (VAS 0–10: 2.8 ± 1.2 vs 2.6 ± 1.0, *p* = 0.420).

**Table-III:** Secondary Outcomes.

Outcome	Onlay (N=48)	Sublay (N=48)	p-value
Operative Time (min)	72.3 ± 12.1	78.6 ± 14.3	0.032
Hospital Stay (days)	4.2 ± 1.1	4.0 ± 0.9	0.318
Postoperative Pain (VAS)	2.8 ± 1.2	2.6 ± 1.0	0.420

## DISCUSSION

This randomized controlled trial demonstrates that sublay mesh repair significantly reduces wound infection rates compared with onlay repair, particularly in patients with larger hernia defects. The lower infection rate in the sublay group (8.3% vs 22.9%, *p* = 0.049) aligns with previous evidence that deeper mesh placement within well-vascularized planes lowers surgical site infection risk.^8^,^12^ In contrast, onlay repair requires extensive subcutaneous dissection, creating dead spaces that predispose to infection and seroma formation.^8^,^9^

Seroma and recurrence rates were comparable between groups, consistent with earlier randomized studies.^8^,^9^,^13^ Stratified analysis revealed that larger hernia defects (>5 cm) were associated with higher infection rates after onlay repair, likely due to increased dissection and larger mesh use. While drainage was not standardized, absence of drains in larger defects may have contributed to fluid collections. Literature suggests selective use of closed suction drainage in extensive dissections can reduce seroma formation without increasing infection risk.^14^

The slightly longer operative time in the sublay group (78.6 ± 14.3 vs 72.3 ± 12.1 minutes, *p* = 0.032) reflects the technical demands of retrorectus dissection.^11^ Despite this, sublay repair showed fewer complications, indicating that improved vascularity and mesh coverage may outweigh the modest increase in operative duration.^4^,^15^ In resource-limited settings such as Pakistan, where laparoscopic facilities are scarce and postoperative wound care is often suboptimal,^16^ sublay repair offers a practical and cost-effective alternative to onlay repair. Its anatomical advantages, reduced tissue trauma, better mesh integration, and lower infection risk make it particularly suitable for such environments.^8^,^15^

The findings partially support the study hypothesis: sublay repair significantly reduced wound infections but showed no significant difference in seroma or recurrence within six months. Studies with longer follow-up have reported higher recurrence rates beyond 12–18 months, underscoring the need for extended surveillance.^15^,^17^ Stratified analysis by defect size and highlights the importance of individualized surgical planning and patient optimization.^5^ Larger defects require bigger meshes and wider dissection, increasing dead space and risk of seroma or infection; this likely explains the higher infection rate with onlay repair for defects >5 cm. Selective placement of drains in large defects may help reduce seroma formation, though evidence remains mixed. Our findings align with Pakistani data demonstrating better outcomes with sublay mesh repair. In a comparable Pakistan prospective study of 80 patients, sublay repair showed lower wound infection rates (5% vs 15%), fewer seromas (4.61% vs 20%), shorter hospital stay, and zero recurrences compared with onlay repair, supporting our findings of reduced wound infection with the sublay technique (8.3% vs 22.9%, *p* = 0.049).).^18^

These results add valuable regional data to global literature, especially for low-resource healthcare systems where laparoscopic options are limited.^19^,^20^ Clinically, the findings support adopting sublay mesh repair as the preferred open technique when performed by trained surgeons, balancing operative efficiency with reduced postoperative morbidity.

### Limitations

This study’s single-center design, small sample size, and lack of blinding may limit generalizability. The six-month follow-up may also be insufficient to detect late recurrences that can appear after 12–18 months.^21^

## CONCLUSION

Sublay mesh repair offers a significant advantage in reducing wound infections while maintaining similar seroma and recurrence rates, making it a preferable option in resource-constrained settings. Its potential cost-effectiveness supports its broader use, though long-term, multicenter studies are warranted to confirm these findings.

### Author`s Contributions:

**SEU:** Conceived and designed the study, Collected clinical data, performed statistical analysis, and contributed to interpretation of results, and is accountable for the overall integrity of the work.

**MIH:** supervised data collection, performed critical revisions.

**IJ:** Drafted the initial manuscript and assisted with literature review.

All authors have read and approved the final version of the manuscript.
